# A qualitative study to explore primary health care practitioners’ perceptions and understanding regarding the COVID-19 pandemic in KwaZulu-Natal, South Africa

**DOI:** 10.4102/phcfm.v13i1.3084

**Published:** 2021-12-08

**Authors:** Celenkosini T. Nxumalo, Gugu G. Mchunu

**Affiliations:** 1 School of Nursing and Public Health, College of Health Sciences, University of KwaZulu-Natal, Durban, South Africa; 2 Faculty of Health Sciences, Durban University of Technology, Durban, South Africa

**Keywords:** COVID-19, coronavirus, coronavirus pandemic, perceptions, understanding

## Abstract

**Background:**

The coronavirus disease 2019 (COVID-19) is a novel virus that has rapidly spread across countries globally, and has been declared a pandemic by the World Health Organization (WHO). In South Africa, more that 1 million cases have been confirmed since case zero was detected in March 2020. South Africa is currently leading in the sub-Saharan African region in terms of COVID-19-related mortality and morbidity rates.

**Aim:**

The aim of this study was to explore primary health care practitioners’ perceptions and understanding regarding the COVID-19 pandemic in KwaZulu-Natal, South Africa.

**Setting:**

The study was conducted at two selected primary health care facilities (a community health centre and satellite clinic) within a low-income rural context in KwaZulu-Natal, South Africa.

**Methods:**

A qualitative study was conducted to explore and describe perceptions and understanding of primary health care practitioners regarding the COVID-19 pandemic in KwaZulu-Natal (KZN), South Africa. Data were collected from a purposive sample of 15 participants at two different clinics situated in rural KZN, South Africa. Participants comprised of nurses, physiotherapists, pharmacists, community care givers, social workers and clinical associates. The participants were both men and women who were all above the age of 20. Data were collected through individual, in-depth face-to-face interviews using a semi-structured interview guide. An audiotape was used to collect data, which were transcribed verbatim. Data were analysed manually by thematic analysis following Tech’s steps of data analysis.

**Results:**

Participants reported pre-pandemic and pandemic perceptions of fear, denial, expectancy and a perceived poor preparation for the COVID-19 outbreak. The findings also revealed participants’ misperceptions regarding the nature of the COVID-19 pandemic and unrealistic expectations of occupational compensations for working during the outbreak.

**Conclusion:**

The findings of this study suggest that primary health care practitioners generally have negative perceptions and understanding regarding the pandemic because of misinformation obtained from social media. Interventions to support health care practitioners are necessary to mitigate the potentially negative implications of health practitioners’ misconceptions on service delivery and their mental health.

## Introduction

In December 2019, the sudden onset of a severe pneumonia-like illness was reported in central China’s Hubei province in the capital city of Wuhan.^1^ Initially, the World Health Organization (WHO) reported it as a cluster of pneumonia cases, the outbreak was later confirmed to be caused by a type of novel coronavirus called ‘severe acute respiratory syndrome coronavirus 2’ (SARS-CoV-2).^2,3^ Infections spread rapidly across China and to other parts of the world, and later, the WHO named the illness as coronavirus disease 2019 (COVID-19); subsequently the spread of the virus was declared a pandemic.^4,5,6^ Following a rapid rise in the incidence, prevalence and associated case fatalities related to SARS-CoV-2, the pandemic was also declared a public health emergency of international concern.^7^

Since December 2019, the virus has spread to more than 200 countries, including South Africa. To date there have been more than 227 million confirmed cases and more than 4.5 million deaths globally.^8^ These numbers are higher than the SARS (8273 cases, 775 deaths) and Middle East Respiratory Syndrome (MERS) (1139 cases, 431 deaths) outbreaks of 2003 and 2013, respectively.^9^ The last time an epidemic of such severity and magnitude was the influenza pandemic of 1918, known as the Spanish ‘flu, caused by the H1N1 virus.^10,11^ The initial spread of COVID-19 in Africa was a combination of sporadic and cluster cases, with the exception of a few countries that have predominantly had cases of travellers and community transmission; one such country is South Africa.^7^

In South Africa, the first case of COVID-19 was reported on the 5 March 2020 in the KwaZulu-Natal (KZN) province.^12^ From the initial report of case zero, there has been a steady rise in the number of confirmed cases together with associated morbidity and mortality. Epidemiological data on COVID-19 show that South Africa currently has the highest number of confirmed cases and mortality in Africa.^13^ Research studies suggest that the heterogenous spatial dimension of the pandemic, nosocomial transmissions and socio-economic circumstances resulted in difficulties to mitigate the spread of the pandemic, thus contributing to higher rates of infection and mortality.^14,15^ Nationally, KZN is rated as the third leading contributor to this burden in terms of mortality rate. As of 15 March 2021, South Africa has recorded more than 2 million confirmed COVID-19 cases, whilst the number of deaths is over 80 000. In KZN, more than 500 000 confirmed COVID-19 cases have been recorded, with more than 14 000 related deaths reported. The number of infections and related deaths continues to increase with every wave of the pandemic and the emergence of new viral strains. In December 2020, a new strain of the causative agent of COVID-19 emerged in South Africa – the former V501.V2 clade, which has been labelled by the WHO as the Beta variant in the classification of COVID-19 variants of concern.^16,17^ This new variant of SARS-CoV-2 appears to be more virulent than the initial strain of the virus and has mutated in several areas of the viral spike protein.^18^ The present COVID-19 variants have been associated with increased transmissibility, virulence and a potential decrease in the effectiveness of certain control measures to curb the spread of the virus.^17^

With the understanding that the R naught (the number of people who will become ill from each infected person) can be heavily reduced, depending on what a nation does to contain the virus, it makes sense that the South African government has implemented several primary prevention interventions to mitigate the spread of the virus. The WHO has estimated the R naught of COVID-19 to be between 1.95 and 2.2, although this differs from country to country.^19,20^ The estimated mortality rate for COVID-19 is approximately 3% globally.^21^ Whilst a variety of factors, such as geography, quality of health care, age of the population, socio-economic factors and underlying conditions, will influence the mortality rate, appropriate interventions targeting these factors can drastically reduce the mortality rate.^22,23^ Whilst the public health and behavioural interventions are what primarily limits the spread of COVID-19, the role of the provision of quality, specialised health care to reducing infection and mortality cannot be understated.

In South Africa, the primary healthcare approach was adopted as a strategy to ensure delivery of comprehensive health care services that are universally accessible to all.^24^ The district health care system comprises of three levels, namely, primary care, secondary and tertiary level care, is the organisational framework that serves as a vehicle for the delivery of the primary health care services.^25,26^

Healthcare workers form the backbone of quality healthcare provision at all levels of healthcare within the national health system. However, research studies on health service delivery have revealed that in the recent years, the quality of healthcare and healthcare delivery has been comprised because of serious staffing shortages.^27,28^ The shortage of staff has been shown to result in burnout, leading to stress, unbearable working conditions and increasing absenteeism, which is accompanied by longer patient waiting times and a general client dissatisfaction with health services, particularly at a primary care level.^29^ The rapidly increasing incidences and morbidity rates associated with COVID-19 infection are thus anticipated to add to the existing burden to the health service-related challenges faced by healthcare workers, particularly within the rural primary care context.

The successful implementation of the COVID-19 national response strategy and other mitigation interventions are heavily reliant on a robust healthcare system, that is adequate, and on appropriate healthcare resources.^30^ In this regard, both policy and clinical practice must be of the highest standard. A review of the current state of COVID-19 management has shown that clinical practice is central to the management of the effects on the disease from a curative, rehabilitative and promotive or preventive perspective.^19^ Since the global outbreak of the pandemic, healthcare practitioners at all levels have been at the forefront of the outbreak in terms of the clinical management of confirmed positive cases of COVID-19.^31^

The initial impact of the pandemic on the quality of life of individuals and communities has highlighted the importance of ensuring that healthcare workers are adequately prepared.^32,33^ Policy development and context-specific operational guidance are core elements in the health systems’ response to COVID-19; they are essential elements in the preparation of healthcare workers for holistic clinical management of the disease to promote health and improve the quality of life across the affected population. Adequate preparation of healthcare practitioners for such an outbreak should extend beyond policy to include clinical training for the holistic care of individuals infected and affected by COVID-19. In addition, healthcare workers should be provided with all the necessary resources to provide health care and be ensured that they are protected from the virus whilst working.^34^

Healthcare workers are at high risk during epidemics.^35^ A review of international literature on previous epidemics has revealed that healthcare workers have experienced numerous challenges, most of which are related to an increased workload because of the clinical demands of patient care.^36,37,38^ Such challenges have had several effects on healthcare workers’ physical and mental well-being and other aspects of their lives. Disease outbreaks can cause not only significant public health concerns but also significant psychological distress, particularly amongst healthcare workers.^39,40^ The known lethality of COVID-19 as well as the intense media coverage of the pandemic can exacerbate perceptions of personal danger. Reports of an increasing number of healthcare workers becoming infected and subsequent deaths can lead to healthcare workers being overwhelmed with feelings of fear and distress.^41^

As a large proportion of the overall SARS outbreaks occurred in hospitals, most studies focus on healthcare workers in the hospital environment.^42,43,44,45^ Primary health care facilities are the first level of entry and come in contact with more community members. A study conducted in Japan, which explored healthcare workers’ perception of risk, knowledge of preventive measures, and perception of infection control measures at the institutional level, found that the concept of institutional measures was the most important predictor of individual perception of risk.^46^ This study also found that most respondents assigned a relatively high importance to hand hygiene and area isolation compared with personal protective equipment. Another study on the risk perception of healthcare workers regarding the Ebola virus in West Africa reported a high prevalence of misinformation amongst voluntary health providers.^35^

In South Africa, particularly in KZN, the outbreak of COVID-19 has led to several healthcare workers becoming infected with the virus, leading to numerous health care facilities almost closed. As South Africa prepares for the implementation of universal coverage and the National Health Insurance (NHI) plan,^47^ primary health care practitioners are at the forefront of the fight against this disease in terms of screening, early detection and initial clinical management of identified cases. In light of the national Department of Health’s COVID-19 response strategy, primary health care practitioners’ roles in the outbreak extend beyond the case finding and management of infected cases to include effective contact tracing and clinical care.^12^ Whilst there have been reports on the negative effects of the outbreak on health care systems, there is little peer-reviewed evidence of health care practitioner experiences, perceptions and understanding of the outbreak. These factors, coupled with the outbreak’s occurrence in low-income rural settings, may add to the existing burden that may be perceived and experienced.^48^ However, there is a paucity of qualitative research data on health workers’ perceptions of COVID-19, particularly in a low-income rural primary healthcare context. Research exploring the reflections of primary healthcare workers regarding COVID-19 is more imperative now than ever.

The aim of this study, therefore, was to explore primary health care practitioners’ perceptions and understanding of the coronavirus (COVID-19) pandemic in KZN, South Africa. This study forms part of a larger study that explores the reflections of primary healthcare practitioners regarding the COVID-19 pandemic in KZN, South Africa.

### Research objectives

- To explore primary healthcare practitioners’ perceptions and understanding regarding the COVID-19 pandemic in KZN, South Africa.
- To explore the perceived level of preparation amongst primary healthcare practitioners regarding the COVID-19 pandemic in KZN, South Africa.

## Methods

### Study design

A qualitative approach using a descriptive design was used to explore and describe primary healthcare practitioners’ perceptions and understanding of the COVID-19 in KZN, South Africa.

### Setting

This research study was conducted at two different primary healthcare facilities (a community health centre and satellite clinic) at a selected health district in KZN, South Africa. The selected primary healthcare facilities are part of the public health sector, which is run by the Department of Health. Both health facilities are situated in a rural area, serving a substantially underdeveloped, under-resourced and largely unemployed community. The facilities offer an array of curative, preventive and promotive healthcare services. The selected health district and primary healthcare facilities were in the near geographical location of the principal investigators, which allowed for a rich immersive understanding of participants’ viewpoints in the context of the community dynamics.

### Sampling and recruitment strategies

Purposive sampling was carried out within the targeted setting to recruit participants to form part of the study. All participants were identified and recruited from within the selected health facilities for data collection. For this study, primary health care practitioners, namely, all categories of permanently employed nurses, social workers, clinical associates, physiotherapists, pharmacists and community caregivers who had at least 6 months experience working at primary care level formed part of the study. These participants had direct contact with both suspected and confirmed COVID-19 patients. Practitioners who were students, volunteers and were not permanently employed were excluded. In this study, the intention was to sample participants from all health disciplines available between the two selected facilities; however, medical doctors, radiographers, dentists, and dental assistance working in the community health centre did not grant consent to participate when recruitment was carried out. Purposive sampling was thus limited to the categories of healthcare workers mentioned. Identification and recruitment of participants were through mediated access, which entailed first informing their immediate supervisors regarding the nature of the study. Supervisors assisted by providing information on participants that would be relevant to the study based on the inclusion criteria of the study. These participants were then approached by the researcher with support from their supervisors, and informed consent to participate in this study was obtained. During the recruitment process, the aim was to identify different categories of healthcare workers who had been exposed to patients who were both suspected and confirmed COVID-19 cases.

### Data collection

A semi-structured interview guide was used to collect data through individual, face-to-face interviews. All interviews were conducted in a private consulting room after participants had completed their clinical duties. All participants had been screened for COVID-19 prior to data collection as part of routine surveillance before they assumed their clinical duties. The safety precautions of COVID-19 were observed by wearing of personal protective equipment (such as surgical masks) by participants and the investigator during data collection. Social distancing within the consulting rooms was also maintained, and strict hand hygiene practices were followed before and after each interview.

A pre-test was conducted with one health care worker prior to commencement of the actual study in order to check the instrument for consistency. No changes were made to the original data collection instrument based on the initial data collected.

The data collection instrument comprised of two sections: The first was related to demographic characteristics of the participants and the second was related to guiding interview questions related to participants’ perceptions and understanding of the COVID-19 pandemic in KZN, South Africa. Data were collected between April 2020 and September 2020 because of the COVID-19 government regulations in effect at the time. All interviews were conducted in English, with a duration of 20–55 min for each interview, and the data were recorded using an audiotape.

### Data analysis

The collected data were transcribed verbatim and then analysed thematically by content analysis. Tesch’s^49^ method of data analysis for qualitative research was used with the following steps: (1) transcripts were read carefully to get a sense of what was contained in them, (2) constant reading and re-reading of the transcript were performed to identify the underlying meaning in the interviews, (3) relevant thoughts and ideas were jotted down in each transcript, (4) this process was repeated with all the transcripts and a list of all topics were made, and (5) similar topics were clustered together to form categories and sub-categories of description in participants’ perceptions and understanding of the COVID-19 pandemic in KZN, South Africa. Data analysis was carried out concurrently with data collection, and recruitment ceased once data redundancy was reached based on the analysis carried out whilst the investigator was collecting the data. Fifteen healthcare workers participated in this study. Data redundancy were achieved by the 12th participant. The extra three participants were added to ensure depth of the collected data.

### Trustworthiness

Data were analysed in collaboration with an expert in qualitative research methodology to ensure trustworthiness. In order to facilitate depth and richness of research findings, individual in-depth face-to-face interviews were conducted. Data analysis began concurrently with data collection, and interviewing ceased once the data redundancy was reached. The data analysis was iterative, encompassing continuous reading and re-reading of the interview transcripts. The transcripts were consistently reviewed and compared with audio-recorded data to ensure the reliability and credibility of the research findings.

## Results

### Socio-demographic data

Fifteen participants who took part in this study included nurses, community care givers, clinical associates, pharmacists, physiotherapists and social workers. All participants were in direct contact with suspected and confirmed COVID-19 cases, and had worked as health practitioners for a minimum of 6 months at a primary healthcare facility. Table 1 shows the demographic details of these participants.

**TABLE 1:** Profile of participants.

Participant	Unique code	Gender	Professional category	Duration working as a healthcare professional	Duration working at a PHC facility	Duration working at current PHC facility	Type of exposure to COVID-19
One	P01	Male	Clinical nurse practitioner	8 years	4 years	4 years	Suspected and confirmed cases
Two	P02	Female	Social worker	10 years	4 years	4 years	Suspected cases
Three	P03	Male	Physiotherapist	8 months	8 months	8 months	Suspected cases
Four	P04	Female	Operational nursing manager	10 years	5 years	3 years	Suspected and confirmed cases
Five	P05	Male	Nutritionist	7 years	4 years	4 years	None
Six	P06	Female	Pharmacy manager	13 years	5 years	5 years	None
Seven	P07	Male	Professional nurse	3 years	2 years	2 years	Suspected and confirmed cases
Eight	P08	Male	Clinical associate	3 years	3 years	3 years	Suspected and confirmed cases
Nine	P09	Female	Community care giver	4 years	4 years	2 years	Suspected and confirmed cases
Ten	P10	Male	Enrolled nurse	5 years	5 years	5 years	Suspected and confirmed cases
Eleven	P11	Female	Enrolled nursing auxiliary	8 years	7 years	7 years	Suspected and confirmed cases
Twelve	P12	Female	Professional nurse	6 years	3 years	3 years	Suspected and confirmed cases
Thirteen	P13	Male	Clinical associate	1 year	1 year	1 year	Suspected and confirmed cases
Fourteen	P14	Female	Community care giver	2 years	2 years	2 years	Suspected and confirmed cases
Fifteen	P15	Male	Professional nurse	18 months	18 months	18 months	Suspected and confirmed cases

COVID-19, coronavirus disease 2019; PHC, primary health care.

Data analysis led to the identification of four main categories of description in participants’ perceptions and understanding of the COVID-19 pandemic in KZN, South Africa. These are as follows: (1) pre-pandemic perceptions (those before the pandemic in South Africa), (2) pandemic perceptions (those when the pandemic occurred in South Africa, (3) perceptions regarding preparedness for COVID-19 and (4) perceived community attitudes towards the COVID-19 outbreak. The four main categories of description yielded 12 sub-categories. Table 2 summarises the main categories of description and their subcategories.

**TABLE 2:** Summary of categories and sub-categories of participants’ perceptions regarding the COVID-19 pandemic in Kwa-Zulu Natal, South Africa.

Category	Sub-category
1. Pre-Pandemic perceptions (those before the pandemic occurred in South Africa)	Fear Denial Expectancy
2. Pandemic perceptions (those when the pandemic occurred in South Africa)	Perceptions regarding the nature and outcomes of the disease Misperceptions regarding COVID-19 Unrealistic expectations
3. Perceptions regarding preparedness for the COVID-19 outbreak	Poor preparation by the Department of Health Lack of personal preparation Government’s response to the pandemic Healthcare workers’ personal expectations
4. Perceived community attitudes towards the COVID-19 outbreak	Positive community attitudes Negative attitudes

COVID-19, coronavirus disease 2019.

### Pre-pandemic perceptions (perceptions before the pandemic occurred in South Africa)

The participants described their perceptions of COVID-19 before the pandemic occurred in South Africa which were mainly influenced by information that they received from social and general media. The participants’ various pre-pandemic perceptions are described in the following sub-categories.

#### Fear

The participants used various terms to express a sense of fear related to COVID-19. This fear stemmed from what participants perceived as the negative social media information and was inherent in the fact that they were unaware of how the disease would eventually manifest if and when it arrived in South Africa. The following statements were made by participants in this regard:

‘It was terrifying in that it included sudden death, and the statistics from other countries as well – so it was frightening then.’ (P01, male, Clinical nurse practitioner)

‘I was so afraid, I thought if this pandemic would come, I became scared for myself and my family, because I know if this thing is airborne, you cannot escape.’ (P04, female, Operational nurse manager)

‘We had already seen from social media how the virus appeared to be killing lots of people so I had a lot of fear and anxiety about what it would do here.’ (P08, male, Clinical associate)

#### Denial

Certain participants believed that the pandemic would be limited to China and would not affect them because China is perceived to be far away from South Africa. Participants also revealed that they did not believe the outbreak was serious because the disease was described as being similar to any normal respiratory illness. This was supported by the following statements:

‘I just did not see it coming, you know. It is just one of those things.’ (P02, female, Social worker)

‘We really did not think it was going become a pandemic. It had not been ruled as a pandemic as of yet.’ (P03, male, Physiotherapist)

‘I did not really think it would occur or spread all over the world because it was said to have originated from a seafood market in China hence, I thought it would stay there so I did not pay much attention to it.’ (P12, female, Professional nurse)

#### Expectancy

Some participants also expressed a sense of foreknowledge that the pandemic would eventually occur in South Africa because of the high rate of outbreaks in neighbouring and other countries further away. These participants revealed that this perception was validated when travel history was cited as a diagnostic criterion testing of COVID-19 in certain affected countries. These participants’ sense of knowing was accompanied by a sense of fear related to the disease because of the perceived complications depicted in social and general media. Participants in this instance were expecting the worst in terms of the effects of the disease when it eventually arrived in South Africa. The following statements support this sub-category of description:

‘My first impression was, it was just a matter of time before we had the case, because looking at the statistics in China, there were other countries that had reported cases. So, really, subconsciously I knew that it is coming to South Africa.’ (P06, female, Pharmacy manager)

‘I suspected that it could end up coming to South Africa, like the other one that was here that affected the flesh …’ (P07, male, Professional nurse)

‘It seemed imminent that it was going to come to South Africa, one way or the other.’ (P08, male, Clinical associate)

### Pandemic perceptions

Participants described their perceptions of COVID-19 when the pandemic occurred in South Africa which appeared to be shaped by an array of factors related to the information they received from various sources, such as the general media, social media, health circulars and peer-to-peer word-of-mouth. These perceptions were also shaped by primary and secondary experiences related to the occurrence of the pandemic in South Africa together with the health systems’ response to the pandemic. The pandemic perceptions are highlighted in the following sub-categories:

#### Perceptions regarding the nature and outcomes of the disease

Participants described their view of the pandemic in terms of the clinical manifestation and ultimate complication of the disease, which they perceived to be a severe disease and that would ultimately lead to death. Certain participants also compared the COVID-19 outbreak with the other outbreaks such as the Ebola virus and H1N1 (commonly known as swine flu). The perception regarding COVID-19 as being deadly was a predominant perception stemming from information that had been circulating on social media before the outbreak occurred in South Africa. This was supported by the following statements:

‘When they talked about it, they talked about it as if it was similar to H1N1 [*swine flu*], which is the swine flu, right? it was the swine flu, mode of spread being, the mode of spread being cough droplets, yea, something of that nature. But, it was more defined as if it was a certain type of COPD [*chronic obstructive pulmonary disease*] or severe, or presented as some kind pneumonia and it was very deadly.’ (P05, male, Nutritionist)

‘I knew it was a viral infection that was caused by the COVID-19 virus [*coronavirus disease 2019*]. It was first identified in China. Yeah and that it spreads through respiratory droplets so like easily transmitted.’ (P08, male, Clinical associate)

‘I understood it to be a contagious viral flu-like illness that appeared to be strangely deadly because of the number of people that were sick and dying in countries like China and Italy as a result.’ (P12, female, Professional nurse)

#### Misperceptions regarding coronavirus disease 2019

Healthcare workers in this study revealed that they had a distorted view of the disease. They reported that their views were related to myths about the disease that had been circulating on social media, which created and perpetuated the notion that the COVID-19 disease was not an outbreak but rather it was manufactured in a laboratory for killing people in order to control population density. Other misperceptions were related to beliefs about power relations between the governments of different countries. This sub-category was supported by the following statements:

‘Initially I also thought that this COVID-19 [*coronavirus disease 2019*] was just a scam to hide what was really going on, there was so much being said by peoples and social media regarding 5G [*5th generation*] and that China wanted to have control over it so they developed this pandemic as a cover up and a means to control nations.’ (P15, male, Professional nurse)

‘I thought that COVID-19 [*coronavirus disease 2019*] was something that was developed for population control, there was even this video saying that COVID [*coronavirus disease*] was a means to kill people because there were already too much people in the world.’ (P13, male, Clinical associate)

‘There were myths about it, that it is unrealistic, it is just flu.’ (P01, male, Clinical nurse practitioner)

#### Unrealistic expectations

Certain participants revealed that whilst they were aware that the pandemic had arrived in South Africa, they did not believe that infection rates would spread as rapidly as they did. They also believed that the disease would be brought under control because only a few cases had been detected in the early days of the outbreak compared with other countries, which was supported by the following statements:

‘I did not really think it was going to be huge. I just thought it was something that was going to die down.’ (P02, female, Social worker)

‘It was a shock really, but also, I knew that eventually we will have it, but I never expected that it would be so fast.’ (P06, female, Pharmacy manager)

‘I didn’t think we would have a problem with the pandemic since our cases were initially low at the start of the pandemic compared to China, America and all those other first world countries. Since Africa was able to fight Ebola which was also really bad I also thought as a country we would quickly find a cure and it would all be over.’ (P13, male, Professional Nurse)

### Perceptions regarding preparedness for coronavirus disease-2019

Participants shared their perspectives regarding the preparation for the COVID-19 outbreak; these perspectives were related to personal preparation prior to the outbreak in South Africa and perceived governmental preparation for the outbreak. The following sub-themes highlight this descriptive category:

#### Poor preparation by the Department of Health

Participants in this study described an array of experiences, which indicated that they perceived the Department of Health to have been underprepared in responding to the pandemic in South Africa. The perceived poor preparation reported by the healthcare workers in this study was also because of the lack of basic essential resources required to enable clinicians to both care for clients and protect themselves whilst providing care to patients. The following statements support this sub-category of description:

‘There was nothing at all. As a result, we were supposed to be prepared by all PPE [*personal protective equipment*] gear, but there was nothing at all. Even the PPE that we were using like masks, it was not surgical masks. It was paper masks, which were very poor.’ (P04, female, Operational nurse manager)

‘When it comes to planning after seeing that something is happening in China and it is spreading to other countries, there was no planning to prepare for when it comes to South Africa.’ (P06, female, Pharmacy manager)

‘I think the government was very late in terms of banning international travel to those countries that were affected. They also responded late in terms of screening and isolation of suspected cases which mean they might have not been adequately prepared despite having verbalised being ready.’ (P09, female, Community care giver)

#### Lack of personal preparation

Health care workers in this study revealed that they personally lacked the preparation needed for the COVID-19 outbreak both from a clinical perspective and as ordinary members of the community. This perception stemmed from their personal views of and experiences with the national health system. The media’s portrayal of failing health systems because of the COVID-19 outbreak was another reason for health workers’ perceived sense of a lack of preparedness. Reporting on this, healthcare workers felt unprepared to face the outbreak in South Africa:

‘We were not trained or in serviced on COVID-19 [*coronavirus disease 2019*] or how to manage a patient with this disease all we knew was what was being revealed by media, even the department did not do any formal training before the pandemic came to South Africa so our knowledge and skill were limited until the pandemic occurred in South Africa.’ (P07, male, Professional nurse)

‘I do not think the government did enough to give the department sufficient funding to take care of the public and its employees. This thing is called COVID-19 [*coronavirus disease 2019*]. It first emerged in 2019. There should have been prior planning at the higher levels of government. As a result I personally felt that we were not ready for COVID-19 as clinician and the entire health system as whole.’ (P09, female, Community Care Giver)

‘I personally felt that healthcare workers were not ready for the pandemic because even when new nurses were employed it was those who had been unemployed for years and staff nurses who were translated to professional nurses and let’s face it how much did they know about health or the pandemic at that time?’ (P14, female, Community care giver)

#### Governments response to the pandemic

Some participants revealed that whilst they thought the government was inadequately prepared for the outbreak, they were of the view that the government’s tactical response to the pandemic was, to a certain degree, adequate based on the nature of the country in terms of the economic and political climate. Other participants felt that the government could have done more to curb the spread of the virus. These participants also felt that the government did not act swiftly enough to halt the spread of new infections, which was supported by the following statements:

‘We responded as best as we could. Let me say that. We are still trying to work as best as we can.’ (P03, male, Physiotherapist)

‘I do not think enough precaution was taken or enough steps were taken to prevent or to make sure that enough has been done to prevent people from getting easily infected, screening people at the gate, sitting arrangements for people, you see. I do not think enough was done to address that.’ (P05, male, Nutritionist)

‘They should have had a way of screening people that are entering the country which is better than the one that they started using which focused on high body temperature only.’ (P07, male, Professional nurse)

#### Healthcare workers personal expectations

Certain study participants revealed their personal expectations from the national government in terms of occupational compensation considering the deadly nature of the pandemic. These healthcare workers reported that whilst they understood the nature of their work as healthcare practitioners, they expected their employer to provide additional compensation because of the unique nature of this pandemic and the hazardous working environment in terms of their personal health and safety. The following excerpts serve to support this notion:

‘Like I thought the State would alleviate us tax-wise or, I do not know, but I thought that would happen, just to better incentivise us to work because at the end of the day we are being asked to put our lives at risk, which is not something we signed up for.’ (P02, female, Social worker)

‘In the forefront of the pandemic and it really put their lives at risk. I think if perhaps there was some kind of compensation or allowance for people that are working during the pandemic, looking at the risks. There is a risk allowance that is available, right?’ (P06, female, Pharmacy manager)

‘The State should do better to recognise our efforts, and not just be like, we commend, and not even mention all the healthcare workers, but just say, “we commend all the doctors and nurses.” I do not like that statement. Show us how you commend us. Alleviate tax. Do something. Give us the 1%. Do something.’ (P10, male, Enrolled nurse)

### Perceived community attitudes regarding the COVID-19 outbreak

Study participants revealed their perspectives regarding patient and community attitudes and behaviours during the COVID-19 pandemic. The perceived community attitudes are highlighted in the sub-categories below:

#### Negative attitudes and behaviours

Certain participants revealed that the community had negative ideas about the origin and course of the disease, and negative attitudes stemming from myths and misconceptions about the COVID-19 pandemic. The participants revealed that their perception of negative community attitudes was manifested in blatant refusals to follow the principles of prevention, such as the wearing of face masks and social distancing, which found validation in the following statements:

‘When it first came people did not really want to adhere to wearing masks, such things, and they had their beliefs that this is not for them.’ (P02, female, Social worker)

‘Some patients still seem to not want to accept that COVID-19 [*coronavirus disease 2019*] is a reality, some think that COVID-19 is a disease for a specific colour and class of people and not them.’ (P11, female, Enrolled nursing auxiliary)

#### Positive attitudes and behaviours

Some participants felt that patients and community members had a positive mindset about the occurrence of the pandemic, in that they witnessed members of the community who understood the COVID-19 pandemic for what it truly is from a biomedical perspective. These community members had been adhering to recommended behavioural interventions to curb the spread of the virus:

‘I am seeing a positive attitude towards the community and understanding the importance of wearing masks and social distancing.’ (P01, male, Clinical nurse practitioner)

‘I think that our patients have been very understanding and compliant with all the changes that have been effected in line with COVID-19 [*coronavirus disease 2019*].’ (P01, female, Pharmacy manager)

## Discussion

The findings of this study revealed four major categories and 12 sub-categories of description in primary healthcare practitioners’ perceptions and understanding of COVID-19 in KZN, South Africa. The first category of description was pre-pandemic perceptions (those before the outbreak occurred in South Africa), and its sub-categories included: fear, denial and expectancy. The second category of description was pandemic perceptions (those when the pandemic occurred in South Africa), and its sub-categories included: perceptions regarding the nature and outcomes of the disease, misperceptions regarding COVID-19, and unrealistic expectations. The third category of description was perceptions regarding preparedness for the COVID-19 outbreak, and its sub-categories were: poor preparation by the Department of Health, lack of personal preparation, and government’s response to the pandemic. The fourth main category was perceived community attitudes regarding the COVID-19 outbreak, and its sub-categories included: positive community attitudes and negative community attitudes. The findings of this study revealed that the perceptions and understanding of COVID-19 amongst primary healthcare practitioners were generally negative. These findings have important implications for clinical practice in terms of pandemic preparedness and ultimately the quality of health care rendered.

Pre-pandemic perceptions of fear, denial and expectancy were reported by study participants. Fear and expectancy were found to be mainly because of the information that participants had received from social media regarding the clinical outcomes of COVID-19. Similarly, high levels of concern were reported by healthcare workers in a recent study to assess the perceptions and attitudes of healthcare workers with regard to the COVID-19 pandemic.^50^ An earlier study that assessed the level of concern amongst hospital-based healthcare workers regarding the MERS outbreak in Saudi Arabia also found high levels of reported concern and risk perception amongst healthcare workers, which could be attributed to previous experience with the outbreaks and the related cultural issues.^51^ The perception of fear can result in adverse mental health outcomes in that it may precipitate behavioural disorders and negative psychological reactions, such as depression and anxiety.^52,53,54^ This may compromise health practitioners’ levels of job satisfaction, which can, in turn, impair their productivity. In this regard, interventions to address healthcare workers’ concerns remain crucial to mitigating the negative impact of such concerns on health service delivery.

This study also found that participants had pre-pandemic perceptions of denial, which could have served as a coping mechanism to deal with anticipated fears about the pandemic. In line with the findings of this study, a recent study on the psychological experiences of COVID-19 patients during hospitalisation found that patients also exhibited attitudes of fear, denial and stigma during the early stages of the disease.^55^ Similarly, Agha^56^ reported denial and avoidance to be the most commonly used coping mechanisms during the lockdown phases of the COVID-19 pandemic. Denial as a coping mechanism was widespread in the initial stages of the outbreak in Wuhan, China.^57^ The use of denial as a coping mechanism highlights the deep-rooted psychological effects of the COVID-19 pandemic. Interventions are, therefore, necessary to assist healthcare workers and the general public to inculcate positive coping mechanisms in order to prevent negative mental health outcomes.^58,59^

Participants in this study revealed pandemic perceptions that were predominantly rooted in a general misunderstanding and misperception about the nature of COVID-19. Similar findings were reported by Bhagavathula et al.,^60^ who cited poor knowledge levels and discrepancies in perceptions of COVID-19 amongst healthcare workers. In another study conducted in Pakistan, healthcare workers were found to have good knowledge; however, there were gaps in specific areas of knowledge, which warranted attention.^61^ It is postulated that misconceptions are deeply rooted in the mindset of healthcare workers, and thus, require targeted interventions that promote continuous professional development of health practitioners.^62^

Study participants had a generally negative perception of the disease process stemming from the information they received from social and general media. This finding is significant as it highlights the important role that social media plays in disseminating information in the present digital age, and also how that information subsequently shapes the attitudes and responses of health-care workers in the fight against the pandemic.^63^ According to recent data, a significant number of healthcare workers and the general population have obtained information about the pandemic from social media, which has been found to present misinformation at an alarmingly high rate.^64,65,66^ Therefore, interventional measures to address unverified and non-scientific information that is shared on social media are needed.^67^ However, social media can also be a valuable tool for rapid dissemination of key knowledge in order to ensure health promotion and citizen engagement in pandemic crisis management.^42,68^

Unrealistic expectations about the course of illness and its impact on communities were also found and were related to the initial trajectory of infection rates in South Africa during the initial period of the outbreak. The review of epidemiological data on COVID-19 in South Africa at the onset of the outbreak showed lower levels of mortality and morbidity when compared with other communicable and non-communicable diseases affecting the country.^69^ Initially, low COVID-19 infection rates and the subsequent finding of unrealistic expectations that are reported in this study are significant as they highlight the possible gaps in the disaster preparedness and disaster response strategy of health systems. The current COVID-19 incidence rate compared with the initial rates of infection bears testimony to this.

Healthcare workers in this study generally perceived government and themselves to be poorly prepared for the outbreak of COVID-19. They reported several negative incidents that supported this view. The shortage of essential resources, such as personal protective equipment and the perceived failure of government to take decisive action timeously to halt the spread of the virus, were some of the descriptive accounts provided by participants to support their notion of poor preparation for the outbreak. Similar findings were cited in a study conducted in Nepal where the majority of participants had negative perceptions of the government’s COVID-19 response and were subsequently unwilling to work during the COVID-19 pandemic.^70^

Whilst there were participants who expressed their dissatisfaction with the government’s response to the pandemic, certain participants perceived the government’s interventions were adequate in light of the contextual factors that influence the country’s system of governance and the general availability of all types of resources.

It was interesting to note that certain participants in this study had personal occupational expectations from their employer in terms of working during the COVID-19 pandemic. These participants mentioned that they expected compensation in the form of bonuses and tax reductions. This would act as an incentive for them to work as frontline healthcare workers, especially given all the challenges they faced regarding their personal protection. Contrary to this finding, Imai et al.^71^ found that providing healthcare workers with a sense of protection motivates them, thus reducing the likelihood of hesitation to work. Conversely, it has been argued that the provision of both financial and non-financial incentives can maximise health care workers’ motivation, particularly in developing regions like Africa.^72^

Participants’ perceptions regarding community behaviour and attitudes to the pandemic were both positive and negative. Their perceptions were related to their experiences with community members in relation to compliance with regulations and behavioural interventions to curb the spread of the pandemic at individual and community levels. A study of the knowledge, attitudes and practices of adults towards COVID-19 in Iran revealed generally appropriate practices amongst the adult population.^73^ These findings concur with the positive perceptions and behaviour reported by certain participants in this study. Similar findings were reported in countries, such as Egypt, Indonesia, and China.^74,75,76^ Conversely, negative perceptions of behaviour were also reported in this study, which are similar to the findings of studies conducted in Nigeria, Pakistan, Bangladesh and the United States where there was a reported practice of poor adherence to behavioural interventions, such as social distancing and hand hygiene.^77,78,79^ These findings highlight the importance of providing tailored information based on the different contextual factors that influence health behaviour.

Support for primary health care practitioners in the form of tailored clinical education and training is essential. The findings of this study also highlight the importance of facilitating the formation of active surveillance and disease monitoring systems at a primary health care level in order to ensure that health care providers are prepared for future outbreaks and pandemics.

## Conclusion

The findings of this study reveal that perceptions and understanding regarding COVID-19 amongst primary healthcare practitioners were mainly centred on a perception of fear, which was related to both known and unknown factors regarding the outcomes of illness associated with the pandemic. The findings also reveal that participants’ perceptions of the pandemic were mainly associated with misinformation received through social media. Overall, the findings of this study reveal that primary healthcare practitioners’ perceptions and understanding of COVID-19 are generally negative. This is cause for concern because it implies that these healthcare workers may be unable to provide the optimum quality of healthcare services as required at a primary care level. Furthermore, these healthcare workers are most at risk for developing mental health issues because of the lack of accurate information regarding the outbreak. This could potentially compromise health practitioners’ levels of job satisfaction potentially disrupting service delivery.
